# The Effect of Scaffold Modulus on the Morphology and Remodeling of Fetal Mesenchymal Stem Cells

**DOI:** 10.3389/fphys.2018.01555

**Published:** 2018-12-21

**Authors:** Abdul Jalil Rufaihah, Suganya Cheyyatraivendran, Muhammad Danial Mohd Mazlan, Kenrich Lim, Mark Seow Khoon Chong, Citra Nurfarah Zaini Mattar, Jerry Kok Yen Chan, Theodoros Kofidis, Dror Seliktar

**Affiliations:** ^1^Department of Surgery, Yong Loo Lin School of Medicine, National University of Singapore, Singapore, Singapore; ^2^Division of Bioengineering, School of Chemical and Biomedical Engineering, Nanyang Technological University, Singapore, Singapore; ^3^Department of Obstretics and Gynaecology, National University of Singapore, Singapore, Singapore; ^4^Department of Cardiac, Thoracic and Vascular Surgery, National University Heart Centre Singapore, National University Health System, Singapore, Singapore; ^5^Nanoscience and Nanotechnology Initiative, National University of Singapore, Singapore, Singapore; ^6^Faculty of Biomedical Engineering, Technion – Israel Institute of Technology, Haifa, Israel

**Keywords:** hydrogel, scaffold, tissue engineering, matrix stiffness, biomaterials, PEGylated fibrinogen

## Abstract

Hydrogel materials have been successfully used as matrices to explore the role of biophysical and biochemical stimuli in directing stem cell behavior. Here, we present our findings on the role of modulus in guiding bone marrow fetal mesenchymal stem cell (BMfMSC) fate determination using semi-synthetic hydrogels made from PEG-fibrinogen (PF). The BMfMSCs were cultivated in the PF for up to 2 weeks to study the influence of matrix modulus (i.e., cross-linking density of the PF) on BMfMSC survival, morphology and integrin expression. Both two-dimensional (2D) and three-dimensional (3D) culture conditions were employed to examine the BMfMSCs as single cells or as cell spheroids. The hydrogel modulus affected the rate of BMfMSC metabolic activity, the integrin expression levels and the cell morphology, both as single cells and as spheroids. The cell seeding density was also found to be an important parameter of the system in that high densities were favorable in facilitating more cell-to-cell contacts that favored higher metabolic activity. Our findings provide important insight about design of a hydrogel scaffold that can be used to optimize the biological response of BMfMSCs for various tissue engineering applications.

## Introduction

Mesenchymal stem cells (MSCs) are multipotent stromal cells which differentiate into multiple connective tissue lineages (osteoblasts, chondrocytes, and adipocytes) under permissive stimulation both *in vitro* and *in vivo* (Krebsbach et al., 1999; Zhang et al., 2010). Adult MSCs are readily isolated from the bone marrow and are able to retain their multipotent differentiation capacity while expanding through multiple passages (Zhang et al., 2010). Recent studies have shown that adult MSCs can be differentiated into specific cells under defined growth conditions or biophysical stimulation (Caplan, 2015). For example, adult MSCs can differentiate into endothelial-like cells after treatment with vascular endothelial growth factor (VEGF) (Oswald et al., 2004). This ability further substantiates their potential to be applied for therapeutic purposes such as in tissue repair and regeneration, where a specific cell lineage is required (Miao et al., 2006). In previous studies, the application of adult MSCs in bone injuries such as calvarial or femoral defects accelerated and improved healing in small and large animal models (mouse, rat, and ovine) (Petite et al., 2000; Cowan et al., 2004; Meinel et al., 2006). However, the main drawbacks of using adult MSCs for tissue repair are the additional trauma associated with the cell harvest, the likelihood that harvesting will yield substantially fewer cells than required for the therapy, and the inevitable need to substantially expand the harvested MSC populations (Redondo et al., 2017). Other limitations of adult MSCs include the inherent heterogeneity of the cell source as well as the age and medical condition of the donor (Redondo et al., 2017). Heterogeneous cell populations require enrichment of the multipotent cells. MSCs of older or chronically sick donors are hard to obtain from the bone marrow and are subjected to age-related decreases in potency (O’Donoghue and Chan, 2006; Zhang et al., 2010). Alternative sources of multipotent cells are sought in order to overcome these limitations. One such potential source is fetal tissue cells, or fetal MSCs (fMSCs) (Campagnoli et al., 2001), which have greater self-renewal and differentiation capacity, longer telomeres, greater telomerase activity, and express additional human telomerase reverse transcriptase. fMSCs are also more readily expandable *in vitro*, and senesce later on during their *in vitro* culture when compared to adult MSCs (O’Donoghue and Chan, 2006).

Hydrogels can provide temporary physical support (i.e., scaffolding) for stem cells to attach, grow and differentiate (Seliktar, 2012). Additionally, transplantation of cells (i.e., cell therapy) into damaged or diseased tissues without a physical support has been shown to be much less effective (Fuoco et al., 2012). Previous studies demonstrated that direct injection of cardiomyocytes delivered in PEG-fibrinogen (PF) hydrogels to the damaged heart following myocardial infarction (MI) increased the viability of the transplanted cells and minimized the infarct size as well as increased angiogenesis in the damaged tissue – when compared to injection of cardiomyocytes without a hydrogel scaffold (Shapira-Schweitzer et al., 2009). Hydrogels can also provide a biomimetic niche to enhance stem cell attachment, proliferation, and differentiation (Nguyen and West, 2002; Naito et al., 2013; Redondo et al., 2017). A major hindrance in the clinical application of stem cells is this ability to guide cell differentiation to specific lineages (O’Donoghue and Chan, 2006). Indeed, as fetal stem cells are multipotent, they could potentially differentiate along an undesired pathway (Chan et al., 2007), whereas the therapeutic effects are contingent upon efficient differentiation along the desired lineage (Chan et al., 2006; Kennea et al., 2009).

Numerous hydrogels have been developed with the objective of guiding stem cell differentiation and enhancing the efficacy of stem cell therapy (Naito et al., 2013; Narayanan et al., 2014; Anjum et al., 2016; Hogrebe and Gooch, 2016; Moshayedi et al., 2016). These hydrogels are often categorized based on the origin of their polymeric constituents: synthetic or biological (Seliktar, 2005). Synthetic hydrogels can be synthesized with precise shape, mechanics, and degradation properties; each of these being tailored to the needs of a particular biomedical application. Synthetic hydrogels made from poly (ethylene glycol) (PEG), for example, have been used in tissue engineering, most notably for cartilage applications (Fan and Wang, 2015; Neumann et al., 2016; Wang et al., 2017). These materials have also been used for the expansion of stem cells in bioreactors (Dias et al., 2017). The drawbacks of using synthetic materials such as PEG include lack of cell adhesion motifs on the polymer, which can lead to poor cell survival. Biological hydrogels offer natural biofunctional motifs on the polymer backbone that can enhance stem cell survival by promoting cell adhesion, proliferation, differentiation, and enzymatic activity. A variety of protein-based hydrogels made from collagen, fibrin or silk fibroin have been used for tissue engineering of bone, cartilage and other applications. However, the use of biological materials could give rise to certain problems in tissue engineering, including limited control over mechanical properties, unregulated biodegradation, possible disease transmission and poor reproducibly (Campagnoli et al., 2001).

Semi-synthetic materials have been proposed to overcome limitation of the synthetic and biological hydrogels (Almany and Seliktar, 2005; Dikovsky et al., 2006; Berkovitch and Seliktar, 2017). The semi-synthetic hydrogels consist of both biological and synthetic polymer constituents, often incorporating bioactive molecules into malleable cross-linked synthetic polymer networks (Lutolf and Hubbell, 2005). One group of semi-synthetic hydrogels in particular – those that mimic the extracellular matrix (ECM) – are becoming more common in cell-based therapy and tissue engineering applications. These hydrogels are designed to possess specific functions that regulate cell fate based on known interactions between cells and natural ECM molecules. We have developed a class of ECM-mimetic semi-synthetic hydrogels using combinations of natural denatured fibrinogen and synthetic hydrophilic polymers (Gonen-Wadmany et al., 2007, 2011; Shachaf et al., 2010). These materials can be formed into hydrogels by mild photochemistry in the presence of cells, thus enabling three-dimensional (3D) culture of the encapsulated cells. The cells interact with the materials by virtue of both biological motifs on the fibrinogen and biophysical cues emanating from the structural properties of the matrix. In this system, the structural properties are controlled by the synthetic polymer constituent; higher concentrations of synthetic polymer increase the cross-linking density and thereby alter the matrix modulus.

In this study, we investigated how different degrees of matrix cross-linking in a PF hydrogel influences the behavior of bone marrow fetal mesenchymal stem cells (BMfMSCs) in both two-dimensional (2D) and 3D culture environments. Specifically, we sought to understand how the modulus affects cell morphology, cell metabolism and cell interactions with the matrix. We made materials containing different amounts of PEG-diacrylate (PEG-DA) and similar amounts of fibrinogen; the higher PEG-DA concentrations resulted in stiffer, more crosslinked PF hydrogels. BMfMSCs were cultured on top of, or within the PF hydrogels, and important parameters of these cells were assessed at various time-points. The results provided evidence as to the modulus-dependent behavior of BMfMSCs both in 2D and 3D culture conditions.

## Materials and Methods

### Synthesis of PEG-Diacrylate and PEG-Fibrinogen

The covalent conjugation of bovine fibrinogen (Bovogen Biologicals Pty Ltd., Australia) to the modified PEG-diacrylate (PEG-DA) was followed according to the published protocols (Elbert and Hubbell, 2001; Dikovsky et al., 2006). Briefly, a 7 mg ml^-1^ solution of fibrinogen in 10 mM phosphate-buffered saline (PBS) with 8M urea was prepared with 0.45 mg ml^-1^ tris (2-carboxyethyl) phosphine hydrochloride (TCEP-HCl) (Sigma, United States). The solution pH was adjusted to 8.0 by addition of NaOH. PEG-DA was prepared from Poly (ethylene glycol)-diol (linear PEG-OH, 10 kDa) using the acryloyl chloride method described elsewhere (Elbert et al., 2001). The PEG-DA was dissolved in 10 mM PBS and 8M Urea to give a concentration of 280 mg ml^-1^, and added to the fibrinogen/TCEP-HCl solution at a volumetric ratio of 6:1 (fibrinogen/TCEP-HCl:PEG-DA). A Michael-type addition reaction between the PEG-DA and the fibrinogen cysteines was used to PEGylate the fibrinogen with diacrylate-functionalized PEG, as described elsewhere (Halstenberg et al., 2002; Rizzi et al., 2006). This PEGylation reaction was done for 3 h in the dark inside a mixture vessel with a thermostatic jacket (Lenz Laborglas, Germany) at a temperature of 22.5°C. Immediately afterward, an equal volume of PBS-8M urea was added to the reaction solution for dilution, and then the reaction product was precipitated with the addition of acetone (Aik Moh Paints and Chemicals Pte Ltd., Singapore), at a volumetric ratio of 4:1 (volume of acetone to volume of diluted reaction solution). The PEGylated fibrinogen reaction product that precipitated from the liquid phase was collected by centrifuging the liquid for 5 min at 20 RCF (relative centrifugal force). The supernatant liquid was removed and discarded. The collected precipitate was dissolved in PBS-8M Urea at a 1.8:1 volumetric ratio of PBS-8M Urea to precipitate volume. A tangential flow filtration method was used to purify and concentrate the modified fibrinogen reaction product against 10 mM PBS (ratio of 80:1 v/w PBS to precipitant) down to a concentration of 8–12 mg ml^-1^ using a Centramate cassette (50 kDa MW cutoff, Pall Corporation, Port Washington, NY, United States). The purified solution was passed through a high shear fluid processor (Microfluidics M110-P, United States) and sterile filtered using a 0.2 micron VacuCap 90 filter (Pall Corporation, United States). The fibrinogen concentration of the sterile PEGylated fibrinogen solution was characterized after filtration using a NanoDrop^TM^ 2000 Spectrophotometer (Thermo Fisher, Waltham, MA, United States).

### Preparation of PF Hydrogel and Characterization

Hydrogels were prepared from a PF hydrogel precursor solution, which is comprised of sterile PEGylated fibrinogen solution (8 mg ml^-1^), 0.1% (w/v) sterile Irgacure^®^2959 photoinitiator (Ciba, Switzerland), and varying amounts of sterile PEG-DA cross-linker for controlling the hydrogel stiffness. Cells were introduced to this PF hydrogel precursor solution, and gelation was facilitated by a light-activated free-radical polymerization reaction, according to published protocols (Schmidt et al., 2006). Briefly, 0.1% (w/v) Irgacure^®^2959 photoinitiator was added to the PEGylated fibrinogen from a photoinitiator stock solution of 10% Irgacure^®^2959 in deionized water containing 70% (v/v) ethanol. PEG-DA was added to this solution at different concentrations to increase the hydrogel crosslinking, from a 15% (w/v) stock solution of PEG-DA in PBS, as detailed elsewhere (Singh et al., 2013). Five different stiffness levels of the hydrogels were chosen using additional PEG-DA percentages (w/v) added to the PEGylated fibrinogen solution as follows: 0% PEG-DA (PF, native hydrogel without additional PEG-DA; Composition A), 0.5% PEG-DA (Composition B), 1% PEG-DA (Composition C), 1.5% PEG-DA (Composition D), and 2% PEG-DA (Composition E). The final concentration of fibrinogen in the solution was 8 mg ml^-1^ for all compositions; this was achieved by diluting the sterile PEGylated fibrinogen solution with PBS beforehand.

#### Rheological Characterization

Rheological characterization was done using AR-G2 rheometer (TA Instrument, United States) as described elsewhere (Gonen-Wadmany et al., 2011; Mironi-Harpaz et al., 2012). A PF hydrogel precursor solution of 200 μl was loaded onto a 20 mm diameter parallel-plate geometry. The PF solution was equilibrated for 1 min before being exposed to 365 nm UV light with the intensity of 5 mW cm^-1^ from an Omnicure Series 2000 light source (Excelitas Technologies Corp., Waltham, MA, United States). Shear modulus data from dynamic time-sweeps were collected during the photopolymerization of the PF solution upon activation with the UV light source. At the end of the crosslinking process, the shear loss modulus (G”) and shear storage modulus (G’) were collected.

### Bone Marrow Fetal Mesenchymal Stem Cells

#### Samples and Ethics

Fetal tissue collection for this research was approved by the Domain Specific Review Board of the National University Hospital, Singapore (DSRB/2006/00154) in compliance with international guidelines regarding the use of fetal tissue for research. Informed written consent was obtained from pregnant women for the usage of fetal tissue for research purposes. Fetal gestational age was determined by ultrasonic crown-rump or femur length measurement. Fetal long bones (femur and humerus) were collected for the isolation of BMfMSC after the pregnancy was medically terminated. Samples were collected from pregnancies at 18–22 weeks gestation.

#### Isolation of Bone Marrow Fetal Mesenchymal Stem Cells

BMfMSC were isolated according to published protocols (Chan et al., 2006, 2007). Briefly, the long bones (femur and humerus) were dissected out and muscle tissues were carefully removed; the bones were wiped with 70% ethanol to prevent myoblast contamination. The two distal ends of the long bone were sliced open and cell suspension were obtained by using a syringe needle flushing of phosphate buffer made from 1M monobasic (18.4 ml) and 1M dibasic (31.6 ml) potassium phosphate in 1 l volume of double distilled H_2_O; The solution was injected at one end of the opening and collected at the other end under a 70 μm cell strainer (Biomed Diagnostics, United States). The cell suspension solution was then introduced with Ficoll-Paque (GE Healthcare, United Kingdom), which would separate the mononuclear cells from the red blood cells and plasma. After centrifugation, the upper layer was aspirated and the mononuclear cell layer was obtained at the interphase. The cells collected at the interphase were washed with PBS and centrifuged again, before suspending them with growth medium. The cell suspension was plated on a cell culture flask (Nunc, United States) and incubated in a 37°C incubator with 5% CO_2_. Spindle-shaped adherent cells were recovered from the primary cultures after 4–7 days and non-adherent cells were removed during medium changes every 2–3 days.

### Cell Culture and Formation of Spheroids

BMfMSC were cultured in a flask coated with 0.2% of gelatin, filled with Dulbecco’ Modified Eagle Medium (DMEM), supplemented with 10% fetal bovine serum (FBS), which would be referred to as growth medium hereafter, in a 37°C incubator with 5% CO_2_. The spheroids were made by using AggreWell^TM^800 (STEMCELL Technologies, Vancouver, BC, Canada) according to the standard protocol by the company.

### Bone Marrow Fetal Mesenchymal Stem Cell Characterization

#### Immunophenotype

The BMfMSCs undergo both immunocytochemistry and flow cytometry screening to test for the cluster of differentiation (CD) marker. CD73, CD90, CD105, CD34, and CD45 were the markers tested. All the primary antibodies were purchased from Miltenyi Biotec, Bergisch Gladbach, Germany and corresponding fluorophore-conjugated secondary antibodies were from Life Technologies (now Thermo Fisher Scientific, United States). For flow cytometry: BMfMSCs were thawed in a 37°C water bath and diluted with growth medium before centrifugation at 400 × *g* for 5 min. The cell pellet was collected and suspended with 1 ml of FACS buffer (Sigma-Aldrich, United States) which consist of 1% bovine serum in 2 mM EDTA PBS and aliquoted to 100 μl each in 2 ml eppendorf tubes. In each 2 ml eppendorf tube, 10 μl of antibody (CD marker) was added and incubated in the dark. After 30 min, the cell suspension was centrifuged at 400 × *g* for 5 min to obtain the cell pellet. The cell pellet was washed with PBS and underwent centrifugation twice. The cell pellet was suspended with FACS buffer before being analyzed through the machine. For immunocytochemistry: Monolayer cultured BMfMSCs were fixed with 4% formalin and permeabilized with acetone and methanol at a ratio of 1:1 at -20°C. After the fixation and permeabilization step, the BMfMSC were blocked with 1% bovine serum albumin in 2 mM EDTA PBS at room temperature. The samples were washed with PBS three times and the primary antibody diluted with PBS (1:10 dilution) was added for 1.5 h and incubated at 4°C. The primary antibody was removed and the cells were washed with PBS. The secondary antibody was diluted with PBS (1:100 dilutions) and added for incubation (30 min). The cells were then washed with PBS and imaged with an Olympus FV-100 (Olympus, United States) using imaging software and a 20×/0.45 and 40×/0.45 objective lens.

#### Multipotent Differentiation

For bone differentiation: BMfMSCs were seeded at 2 × 10^4^ cell cm^-2^ in a culture plate with growth medium. After overnight culture, the growth medium was changed to a bone differentiation medium [growth medium supplemented with 10^-8^ M dexamethasone, 0.2 mM ascorbic acid, 10 mM b-glycerol phosphate (all Sigma-Aldrich, United States)] and the medium was changed every 3 days for duration of 2 weeks. Cell changes in shape and the production of calcium salts and phosphates were detected through Alizarin Red (Sigma-Aldrich, United States) and Von Kossa (Sigma-Aldrich, United States) staining, respectively. For adipogenic differentiation: BMfMSCs were seeded at 2 × 10^4^ cells cm^-2^ with growth medium. After overnight culture, the growth medium was changed to fat differentiation induction medium [growth medium supplemented with 5 mg ml^-1^ Insulin, 10^-4^ M Dexamethasone, 6 × 10^-3^M Indomethacin (all Sigma-Aldrich, United States)] and incubated for 3 days. After 3 days, the fat differentiation induction medium was changed to a growth medium and incubated for an additional 3 days. This step was done three times. Lipids vacuoles were visible after 14–21 days and were verified by oil red O (Sigma-Aldrich, United States) staining. For chondrogenic differentiation: BMfMSCs were suspended in chondrogenic differentiation medium [growth medium supplemented with 10 ng ml^-1^ TGF-B3, 100 nM dexamethasone, 50 ug ml^-1^ ascorbic acid, 100 ug ml^-1^ sodium pyruvate, 40 ug ml^-1^ proline and 1x ITS (all Sigma-Aldrich, United States)] at room temperature after being centrifuged twice at 150 × *g* for 5 min. After the third centrifugation of the cells, they were kept in the incubator. Medium was changed every 2–3 days for 25 days. Cell pellets were harvested and fixed before being embedded with formalin and paraffin to undergo sectioning (4 μm thick slices). The samples were deparaffinized and rehydrated before alcian blue staining (Sigma-Aldrich, United States).

### Cell Morphology and Remodeling

For 2D: Both BMfMSCs (7,000 cells) and BMfMSC-spheroids (4,000 cells per spheroid) were seeded on top of polymerized PF hydrogel in a single well of a 15-well μ-Slide Angiogenesis plate (Ibidi GmbH, Germany) before medium was added. For 3D: The precursor PF solution (50 μl, 8 mg ml^-1^) was mixed with BMfMSCs (15,000 cells) or BMfMSC-spheroids and polymerized for 3 min under a UV lamp (365 nm, 4–5 mW cm^-2^) before medium was added. At each time point, at least six hydrogel samples of the BMfMSCs and BMfMSC-spheroids – six spheroids in each sample for both the 2D and 3D – were fixed with 4% paraformaldehyde and permeabilized with 0.1% Triton-X. The cells were stained with Alexa Fluor^®^ 488 Phalloidin (Life Technologies, now Thermo Fisher Scientific, United States) to visualize the arrangement of the actin filaments and counter-stained with Hoechst 33342 (Life Technologies, now Thermo Fisher Scientific, United States) for imaging the nucleus. Images were taken using a Zeiss LSM 700 Laser Scanning Microscope (Carl Zeiss AG, Oberkochen, Germany). Lamellipodia were visually quantified as the thin cytoplasmic sheets that extended at the front of the cells; whereas filopodia were visually quantified as the finger-like projections at the edges of the cells. Quantifications were performed on at least four micrographs for each sample of each treatment and composition.

### Cell Metabolism

Cell metabolism was measured by using an alamarBlue^®^ Assay. Resazurin X100 (Sigma-Aldrich, St. Louis, MO, United States) was dissolved with PBS to give a stock concentration of 10 mg ml^-1^. To obtain the working solution, the dissolved stock solution was further diluted with PBS to give a concentration of 1 mg ml^-1^. After polymerization of the gel, medium and alamarBlue were added to the samples with a ratio of 10:1. At each time point period, the solution was extracted and a fresh batch of medium and alamarBlue were added in. The extracted samples were measured using a NanoDrop^TM^ 2000 Spectrophotometer (Thermo Fisher, Waltham, MA, United States) at two specific wavelengths (570 nm and 600 nm) and the percentage of reduction of alamarBlue was calculated. At least six samples for each composition were assessed for analysis.

### Gene Expression

For both 2D and 3D BMfMSCs and BMfMSC-spheroids, RNA was extracted using ReliaPrep^TM^ RNA Cell Miniprep System (Promega, Madison, WI, United States). The PF hydrogels were degraded with collagenase from *Clostridium histolyticum* (Sigma-Aldrich, St. Louis, MO, United States) at the concentration of 0.001 g/ml for 6 h for 2D and 24 h for 3D. After the isolation of the RNA, cDNA was synthetized using QuantiTect^®^ Reverse Transcription Kit (QIAGEN, Hilden, Germany) and stored at -20°C. Real Time PCR was performed in triplicates using Taqman Universal PCR Master Mix [Applied Biosystems (now Thermo Fisher Scientific), Foster City, CA, United States]) and Bio-Rad CFX96 Thermal Cycler machine as per manufacturer’s instructions to check on the expression of integrin complexes on both 2D and 3D environments. The qPCR protocol was as follows: 10 min heat activation at 95°C. After which, 40 cycles were repeated for the following: 15 s of 95°C and 1 min of 60°C. Primers that were used were purchased from Life Technologies, now Thermo Fisher Scientific, United States. The assay IDs for the primers used are as follows: α5 Integrin, Hs01547673_m1; β1 Integrin, Hs00559595_m1; β3 Integrin, Hs01001469_m1; β3 Integrin ID: Hs01001469_m1; β5 Integrin, Hs00174435_m1. The housekeeping gene 18S was used as a reference (Hs99999901_s1). These primers were reported for use with qPCR in prior studies (Bergmann et al., 2011; Frittoli et al., 2014; Plessl et al., 2015; Raman et al., 2016). All gene expression data was normalization with respect to the expression levels of the composition A group, for each of the respective treatments. The formulae used to normalize the data was the same as reported by Livak and Schmittgen (2001) and Schmittgen and Livak (2008), as follows:

ΔCt=Ct(A)−Ct ref (A)(reference gene is 18S)

ΔΔCt=(Δ Ct of experiment)−(Δ Ct of control)

Fold change = 2ˆ-(ΔΔCt) = this delta delta Ct is the simple formula being used to calculate relative fold gene expression of samples when doing qPCR. At least six samples for each composition were assessed for analysis.

### Statistical Analysis

The quantification analysis of the tube length was done by using WimTube software analysis and all the graphs were plotted using Microsoft Excel. Data was presented as group mean ± standard deviations (SD) which was done using Microsoft Excel software. Comparisons between groups and different time points were done using a one-way analysis of variance (one-way ANOVA) with SPSS Software (V.16). Bonferroni or Games Howell *post hoc* tests were used based on Levene’s Test analysis output. Significance was established at *p* < 0.05.

## Results

### Characteristics and Mechanical Properties of PF Hydrogels

**FIGURE 1:**
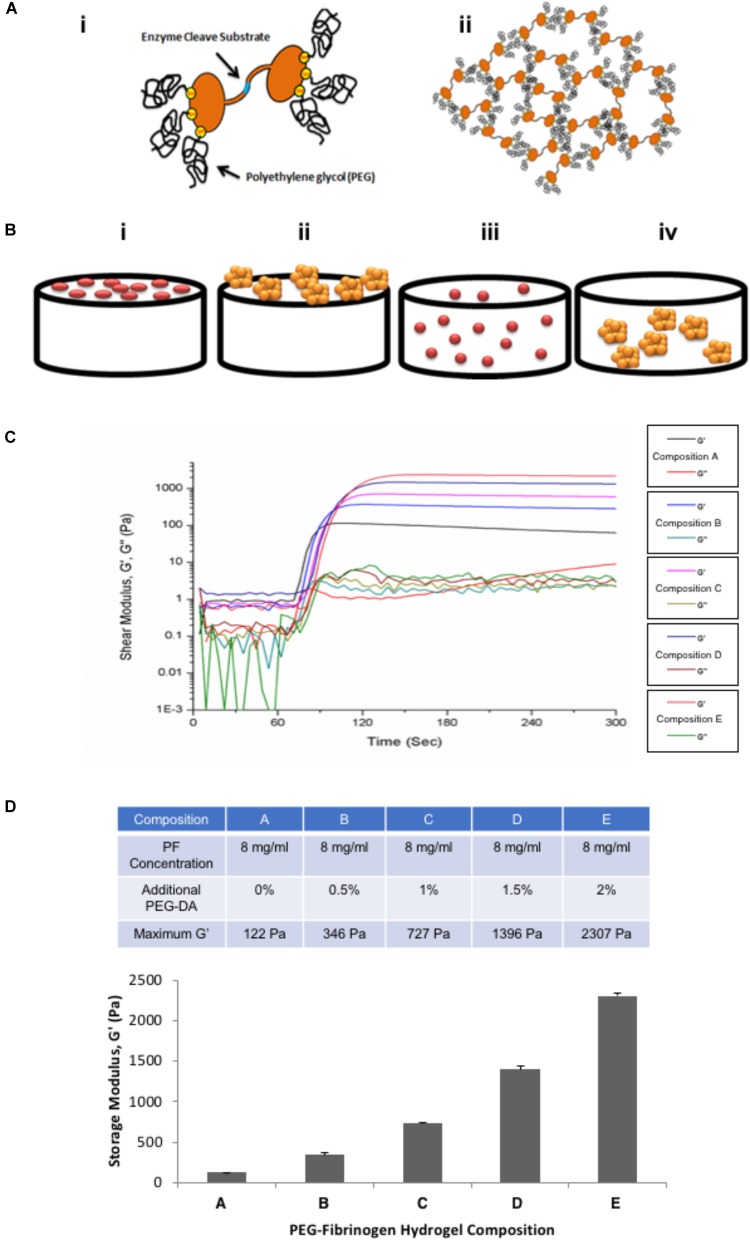
**(A)** Schematic illustration of PEG-fibrinogen (PF) hydrogel assembly. (i) Denatured fibrinogen fragments covalently attach to PEG in order to form a hydrogel precursor with protease cleavage sites (blue) and multiple thiol groups (yellow) which conjugate with functionalized PEG-diacrylate (PEG-DA). (ii) Assembly of PF hydrogels by light-activated radical-polymerization with additional PEG-DA resulting in a cross-linked hydrogel network. **(B)** Schematic illustration of experimental design: (i) BMfMSCs as single cells seeded on the surface of the hydrogel (2D); (ii) BMfMSC-spheroids on the surface of the hydrogel (2D); (iii) BMfMSCs as single cells cultured within the hydrogel (3D). There were six spheroids in each hydrogel for 2D and 3D cultures; (iv) BMfMSC-spheroids cultured within the hydrogel (3D). **(C)** Shear rheometry data from dynamic time-sweep tests performed during the photo-polymerization of the PF hydrogel precursor with photoinitiator upon activation with a long-wave ultra-violet (UV) light source after 60 s; the graph shows the shear storage modulus (G’, Pa) and shear loss modulus (G”, Pa) of various PF hydrogel compositions. **(D)** Graphical representation showing average values of the plateau shear storage modulus of various PF hydrogel as a function of percent additional PEG-DA cross-linker (table shows the average of the maximum storage modulus values of the different hydrogel compositions tested).

Schematic illustrations of the PF hydrogel preparation as well as the experimental design for single cell BMfMSCs and BMfMSC-spheroids seeded on the surface of the PF hydrogels or encapsulated inside the PF hydrogel are shown in Figures 1A,B. The protein concentration in the PF solution was measured to be 10.26 mg ml^-1^ using a NanoDrop spectrophotometer at a wavelength of 280 nm (Rufaihah et al., 2013). During the preparation of the PF hydrogel precursor solution, PBS was added to dilute the fibrinogen concentration to 8 mg ml^-1^ and PEG-DA was added to increase the crosslinking and the material’s maximum shear storage modulus. The mechanical properties with and without the addition of PEG-DA were rheologically analyzed and the crosslinking kinetics are shown in Figure 1C. The maximum values of the shear storage modulus, G’ (Pa), which represented the complete cross-linked state of the PF hydrogel, were significantly affected by the increased concentrations of additional PEG-DA as shown in Figure 1D (*p* < 0.05).

### Characterization of BMfMSCs

**FIGURE 2:**
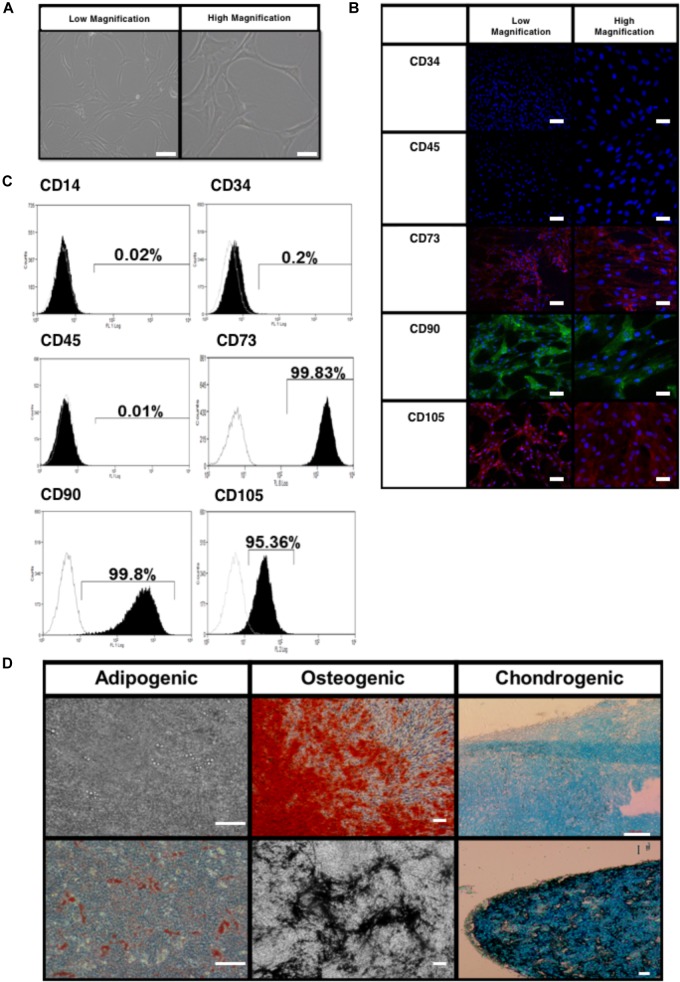
Characterization of bone marrow-derived fetal mesenchymal stem cells (BMfMSC). **(A)** BMfMSCs exhibit a spindled morphology when cultured on tissue substrates (shown are phase contrast low and high magnifications). Immunocytochemistry **(B)** and flow cytometry **(C)** show positive expression of mesenchymal stem cell markers and cells stain negative for hemopoietic markers. Differentiation of BMfMSCs **(D)** was evaluated by Alizarin Red for extracellular calcium (red crystal), von Kossa for extracellular phosphate (black crystal), oil red O for intracytoplasmic lipids vacuoles (red vacuoles) and Alcian Blue stain for extracellular cartilage stains in pellet culture (dark blue). Scale bars: 100 μm for phase contrast low magnification; 25 μm for phase contrast high magnification; 50 μm for flow cytometry low magnification; 20 μm for flow cytometry high magnification; 100 μm for differentiation images.

The BMfMSCs exhibited a spindle shaped morphology when cultured on plastic substrates as seen by phase contrast microscopy (Figure 2A). Immunophenotype of the BMfMSCs were assessed by immunocytochemistry and flow cytometry. Immunostaining was negative for hemopoietic markers (CD34, CD45) and positive for mesenchymal markers (CD105, CD73) and adhesion molecules (CD90) (Figure 2B). Flow cytometry data also confirmed the presence of both mesenchymal and adhesion markers CD105 (95.25%), CD73 (99.57%), CD 90 (99.28%) and the absence of hemopoietic markers CD34 (2.81%) and CD45 (5.62%) (Figure 2C).

The BMfMSCs readily differentiated into adipogenic, osteogenic and chondrogenic lineages under their respective inductive culture conditions. For adipogenesis, BMfMSCs were cultured in adipogenic inductive medium for 21 days. The presence of intracytoplasmic lipid vacuoles was confirmed by oil red O staining (Figure 2D). For osteogenesis, BMfMSCs were cultured in osteogenic inductive medium for 14 days and stained with von Kossa and Alizarin Red staining to detect the secretion of extracellular calcium and phosphate crystals, respectively (Figure 2D). For chondrogenic differentiation, BMfMSC pellets were cultured in chondrogenic inductive medium for 25 days and stained with Alcian Blue Stain to detect the ECM of proteoglycans (Figure 2D).

### Cell Metabolism

**FIGURE 3:**
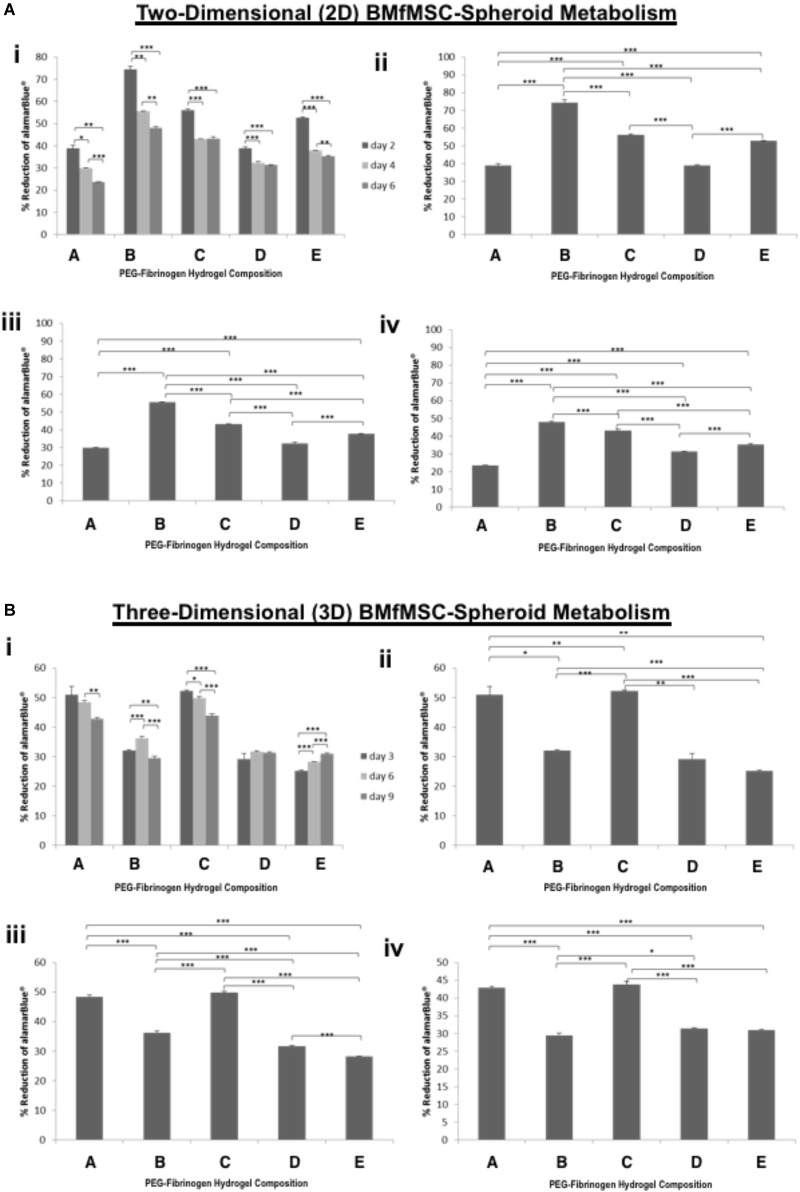
Proliferation of BMfMSC-spheroid cultures in 2D and 3D is affected by PF hydrogel modulus. **(A)** The percent reduction in alamarBlue staining is directly proportional to the cell metabolism of BMfMSC-spheroids seeded on top of the five different compositions of PF hydrogels. The alamarBlue summary for all time-point (i) is broken down for statistical analysis on day 2 (ii), day 4 (iii), and day 6 (iv). **(B)** The percent reduction in alamarBlue for the BMfMSC-spheroids encapsulated in five different compositions of PF hydrogels is summarized for all time-point (i) and broken down for statistical analysis on day 3 (ii), day 6 (iii), and day 9 (iv). Data is expressed as the mean plus/minus standard deviations. Statistical significance between days or compositions was presented by: ^∗^*p* < 0.05; ^∗∗^*p* < 0.01 and ^∗∗∗^*p* < 0.001 (*n* ≥ 6).

**FIGURE 4:**
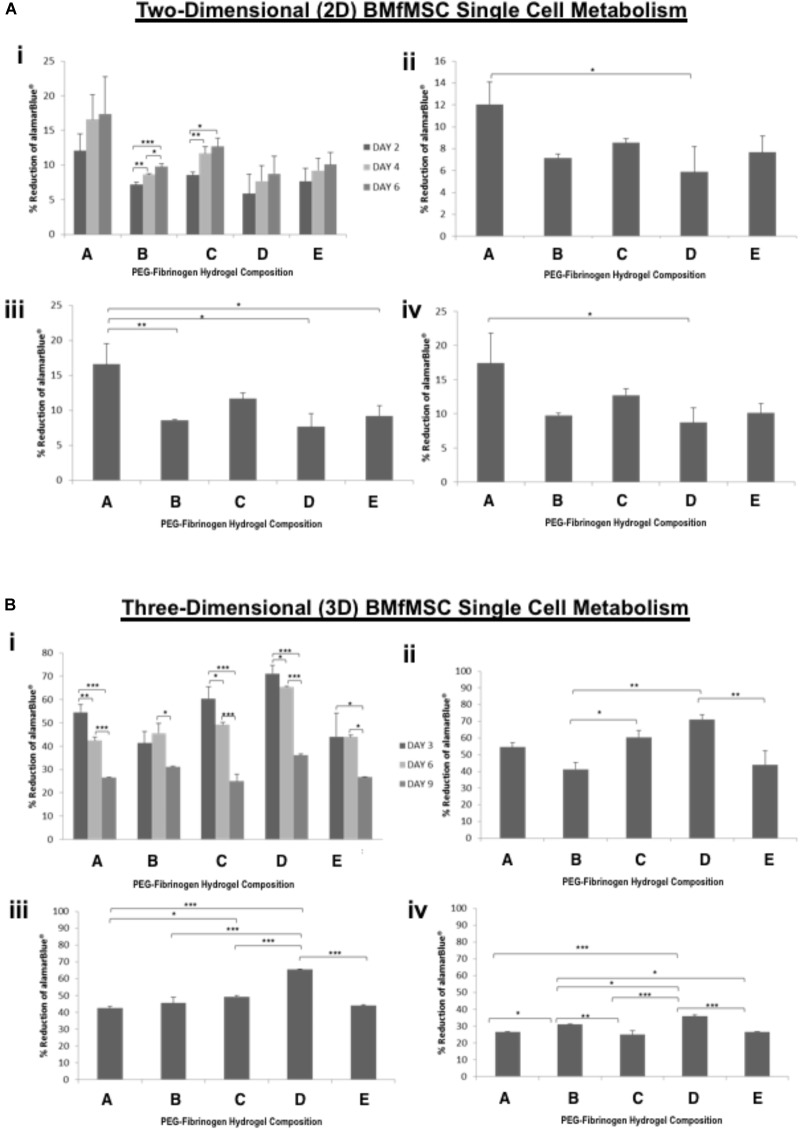
AlamarBlue for BMfMSCs as single cell cultures in 2D and 3D is affected by PF hydrogel modulus. **(A)** The percent reduction in alamarBlue staining is directly proportional to the cell metabolism of BMfMSCs as single cells seeded on top of the five different compositions of PF hydrogels. The proliferation summary for all time-point (i) is broken down for statistical analysis on day 2 (ii), day 4 (iii), and day 6 (iv). **(B)** The percent reduction in alamarBlue for the BMfMSCs as single cells encapsulated in five different compositions of PF hydrogels is summarized for all time-point (i) and broken down for statistical analysis on day 3 (ii), day 6 (iii), and day 9 (iv). Data is expressed as the mean plus/minus standard deviations. Statistical significance between days or compositions was presented by: ^∗^*p* < 0.05; ^∗∗^*p* < 0.01 and ^∗∗∗^*p* < 0.001 (*n* ≥ 6).

The alamarBlue was used as a reagent for evaluating cellular health; it assess whether cells have enough energy to proliferate. The metabolism of BMfMSC-spheroids (Figure 3) and BMfMSC single cell cultures (Figure 4), seeded on the surface of the PF hydrogel (i.e., 2D culture) or encapsulated in the PF hydrogel (i.e., 3D culture) showed different metabolic rates for each respective culture condition at the different time points. For example, there was a significant decrease in metabolic activity in the BMfMSC-spheroids from day 2 to day 6 in 2D culture [Figures 3A(i), B(i)]. In 3D culture of BMfMSC-spheroids, there was a significant reduction in metabolic activity of encapsulated cells in composition A and composition C hydrogels from day 3 to day 9, whereas in composition D and composition E hydrogels, there was a significant increase in metabolic activity from day 3 to day 9 (Figure 3B).

In 2D BMfMSC single cell culture, there was a significant increase in metabolic activity from day 2 to day 6 on composition B and composition C hydrogels (Figure 4A). The metabolic activity of BMfMSC single cell cultures in the 3D PF hydrogel constructs decreased significantly from day 3 to day 9 (Figure 4B), following a similar trend to that of the BMfMSC-spheroids in 2D culture (Figure 3A).

### Cell Morphology and Remodeling

**FIGURE 5:**
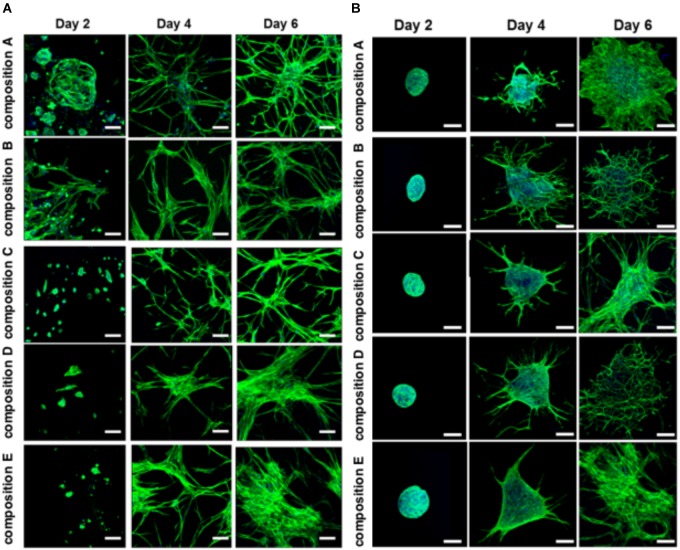
Cell morphology of BMfMSCs as single cell cultures and spheroid cultures on PF hydrogels in 2D at day 2, 4, and 6. Immunofluorescence staining was performed with filamentous actin (f-actin, stained in green) using Alexa Fluor 488 phalloidin and a nuclear counterstain with Hoechst dye (in blue). The BMfMSCs as single cells **(A)** on five different compositions of PF hydrogels show the relationship between composition-dependent modulus and cell morphology. The BMfMSCs as spheroids **(B)** on five different compositions of PF hydrogels also shows the relationship between composition-dependent modulus and cell morphology. Scale bar: 50 μm for single cell images and 100 μm for the spheroid images.

The BMfMSCs as single cells seeded on the surface of PF hydrogels of differing degrees of stiffness displayed both lamellipodia and filopodia starting at day 4 onward (Figure 5A and Table 1). Hydrogels having low levels of PEG-DA crosslinker (compositions A, B, and C) showed more filopodia at day 6, whereas on compositions D and E, the cells showed more lamellipodia. The BMfMSC-spheroids seeded on the surface of PF hydrogels also showed lamellipodia and filopodia from day 4 onward and showed extensive protrusions at day 6 (Figure 5B). The composition A and composition E hydrogels contained cells with more lamellipodia at day 6, whereas composition B and composition D hydrogels contained cells with more filopodia; the composition C hydrogel contained cells that showed similar levels of lamellipodia and filopodia.

**FIGURE 6:**
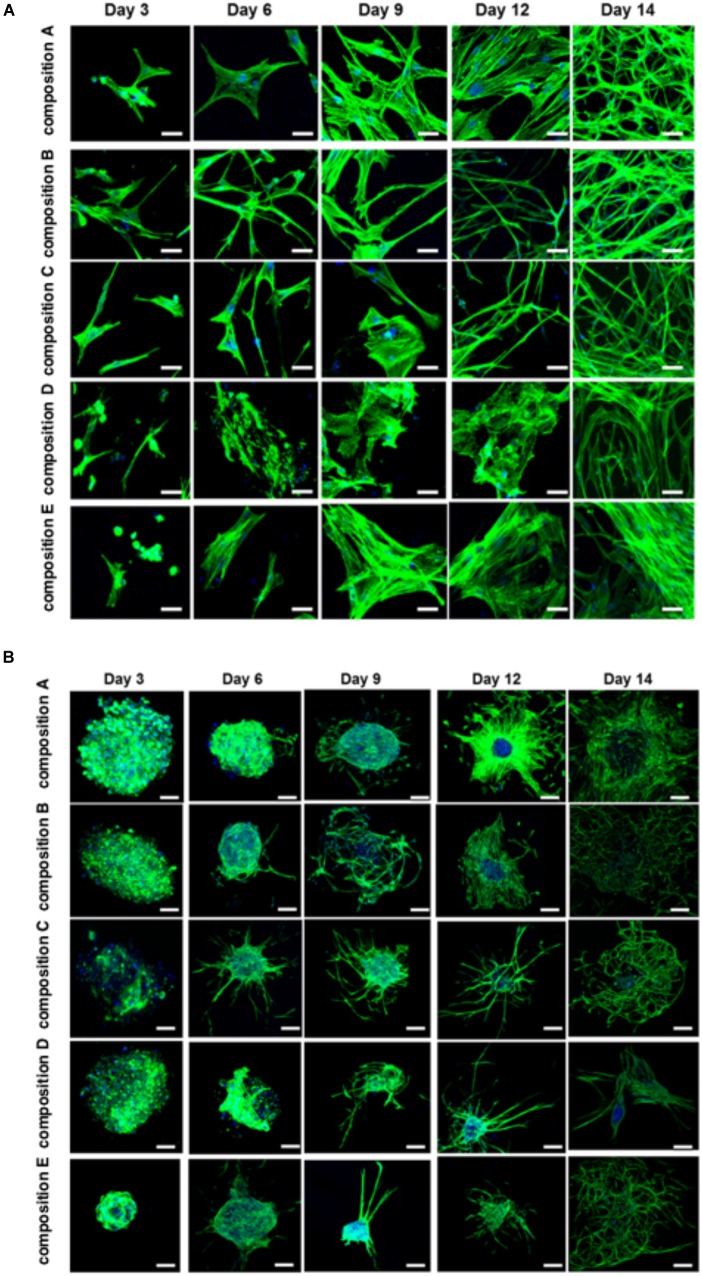
Cell morphology of BMfMSCs as single cell cultures and spheroid cultures in PF hydrogels in 3D at day 3, 6, 9, 12, and 14. Immunofluorescence staining was performed with filamentous actin (f-actin, stained in green) using Alexa Fluor 488 phalloidin and a nuclear counterstain with Hoechst dye (in blue). The BMfMSCs as single cells **(A)** on five different compositions of PF hydrogels show the relationship between composition-dependent modulus and cell morphology. The BMfMSCs as spheroids **(B)** on five different compositions of PF hydrogels also shows the relationship between composition-dependent modulus and cell morphology. Scale bar: 20 μm for the single cell images and 100 μm for the spheroid images.

The BMfMSCs that were encapsulated in PF hydrogels showed lamellipodia and filopodia from day 3 onward (Figure 6A and Table 2). In composition A, composition D and composition E hydrogels, only lamellipodia were observed at day 6, whereas in composition B and composition C hydrogels, a mixture of lamellipodia and filopodia were observed at day 6. The composition D and composition E hydrogels contained cells with extensive protrusions of lamellipodia at day 9 and day 12. The composition D hydrogels contained cells with moderate levels of filopodia and composition E hydrogel contained cells with slight protrusions of the same at day 14. The BMfMSC-spheroids encapsulated in PF hydrogel showed lamellipodia and filopodia from day 6 onward (Figure 6B). The composition A, composition C and composition E hydrogels contained cells presenting similar protrusions of lamellipodia and filopodia at day 9 and the other two hydrogel groups contained cells showing pronounced filopodia protrusions. At day 12, the composition A contained cells with extensive lamellipodia protrusions, the composition B and composition E hydrogels contained cells with equal protrusions of both, whereas the composition C and composition D contained cells with more filopodia protrusions. At day 14, more lamellipodia were observed in cells on the composition A and composition D hydrogels, whereas more filopodia were observed on the composition B composition C and composition E hydrogels.

**FIGURE 7:**
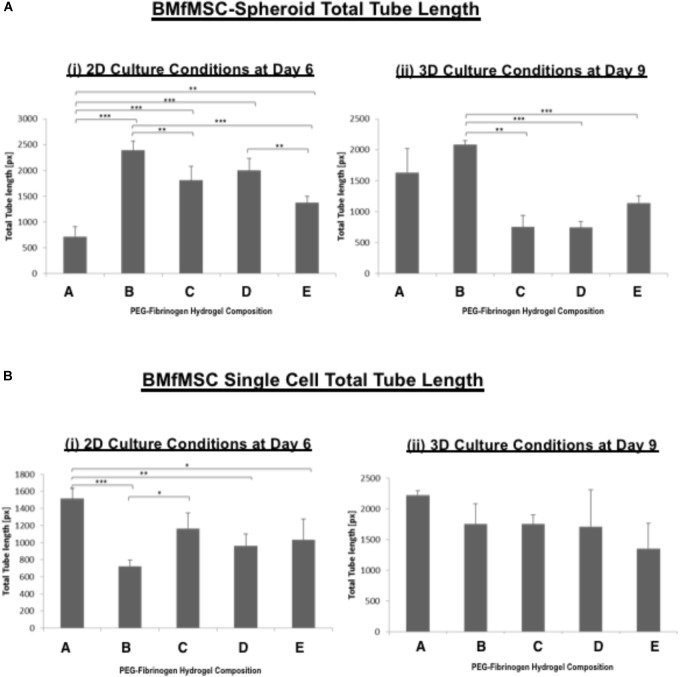
The total tube length of BMfMSCs was affected by the modulus of the PF hydrogel matrix. Quantitative analysis of total tube length for both **(A)** BMfMSCs-spheroids and **(B)** BMfMSC as single cells in 2D and 3D was performed using WimTube software. The BMfMSC-spheroids seeded on top of the PF hydrogels (i) and encapsulated in the PF hydrogels (ii) exhibited differences in tube length for the different compositions of the PF hydrogels. The fMSCs as single cells seeded on top of the hydrogel (iii) and encapsulated within the hydrogels (iv) also showed differences in tube length in the different compositions of PF hydrogels, but these differences were not significant in encapsulated fMSCs as single cells. Data is expressed as the mean plus/minus standard deviation. Statistical significances between each composition was represented by: ^∗^*p* < 0.05; ^∗∗^*p* < 0.01 and ^∗∗∗^*p* < 0.001 (*n* ≥ 6).

The quantitative analysis of total cell tube length using WimTube software showed highly variable results for both single cell cultures of BMfMSCs and BMfMSC-spheroids with hydrogels of varying stiffness in 2D and 3D (Figure 7). The BMfMSC-spheroids in the composition B hydrogel group exhibited significantly more total tube length than the other four hydrogel groups for both 2D and 3D experiments (Figure 7A). For the single cell BMfMSCs in the composition A hydrogel group, the total tube length measured was significantly higher than the other four hydrogels in both 2D and 3D studies (Figure 7B). At least four micrographs for each sample (*n* = 1) were quantified for this assessment; a minimum of six samples were used for the data analysis of each composition.

### Gene Expression

Quantitative reverse transcription PCR (qRT-PCR) results showed different expression of integrin subunits such as α_5_, β1, β_3_ and β_5_ integrin subunits for BMfMSC single cells and BMfMSC-spheroids in different hydrogel groups both in 2D and 3D experiments (Table 3). In the single cell 2D experiments, BMfMSCs on composition D hydrogel expressed an increase in α_5_, β_1_ and β_5_ integrin subunits by 13.5-fold, 16.5-fold, and 3.9-fold, respectively, as compared to the expression levels of BMfMSCs seeded on composition A hydrogel at day 2. The expression levels of that group were drastically reduced by day 4 and too low to be detected at day 6. In the BMfMSC-spheroid 2D experiments, the composition B and composition D hydrogel showed a 12.8-fold and 2.7-fold increase in expression of α_5_ integrin subunit compared to the expression on composition A hydrogels. Although the expression of β_3_ integrin subunit was analyzed, their levels were undetected in both the 2D single cell and the spheroid experiments. A minimum of six samples were used for the analysis of each composition and treatment.

In the 3D single cell experiments, BMfMSCs in composition B hydrogels showed a 14.4-fold increase in expression of β_1_ integrin subunit compared to those in composition A hydrogels at day 3 but the same treatment showed 1.3-fold more expression of β_1_ integrin subunit compared to that of the composition A hydrogel at day 14. The BMfMSCs on the composition E hydrogels showed 20.3-fold, 3.8-fold, 10.9-fold increase in expression of β_1_, β_3_, and β_5_ integrin subunits, respectively, compared to those in composition A hydrogel at day 14.

The BMfMSC-spheroids encapsulated in composition E hydrogel showed a 15-fold and 16.3-fold increase in expression of α_5_ integrin subunit at day 3 and day 6, respectively, compared to those in composition A hydrogels. BMfMSC-spheroids encapsulated in composition E hydrogels showed an 8.1-fold, 12.6-fold, and 20.7-fold increase in expression of β_1_, β_3_, and β_5_ integrin subunits, respectively, compared to those in composition A hydrogel at day 3. At day 6, composition E hydrogels showed a 4-fold, 11-fold, and 19.3-fold increase in expression of β_1_, β_3_, and β_5_ integrin subunits, respectively, compared to those in composition A hydrogel. The expression of β_1_, β_3_, and β_5_ integrin subunits were too low to be detected at the later time points of the experiments (from day 9 to day 12).

**Table 1:** Levels of lamellipodia and filopodia in BMfMSCs as singe cells and as spheroids in 2D, as assessed from immunofluorescence staining (*n* ≥ 6).

Time point	Composition	2D BMfMSCs as single cells		2D BMfMSC-spheroids	
			
		Lamellipodia	Filopodia	Lamellipodia	Filopodia
Day 2	A	-	-	-	-
	B	-	-	-	-
	C	-	-	-	-
	D	-	-	-	-
	E	-	-	-	-
Day 4	A B	+ +	++ ++	+ +	+ ++
	C	+	++	+	+
	D	++	+	++	+
	E	++	+	++	+
Day 6	A	++	+++	+++	-
	B	++	+++	-	+++
	C	++	+++	++	++
	D	+++	+	-	+++
	E	+++	+	+++	+


**Table 2:** Levels of lamellipodia and filopodia in BMfMSCs as singe cells and as spheroids in 3D, as assessed from immunofluorescence staining (*n* ≥ 6).

Time point	Composition	3D BMfMSC as single cells		3D BMfMSC-spheroids	
			
		Lamellipodia	Filopodia	Lamellipodia	Filopodia
Day 3	A	+	-	-	-
	B	+	+	-	-
	C	+	+	-	-
	D	+	+	-	-
	E	+	-	-	-
Day 6	A	+	-	+	+
	B	+	+	+	+
	C	+	+	+	++
	D	+	-	+	--
	E	+	-	+	++
Day 9	A	+	+	+	+
	B	+	+	+	++
	C	+	+	++	++
	D	+++	-	+	++
	E	+++	-	+	+
Day 12	A	+	+	+++	+
	B	-	++	+	+
	C	-	++	-	++
	D	+++	-	+	++
	E	+++	-	++	++
					
Day 14	A	+	+++	+++	+
	B	+	+++	-	+++
	C	+	+++	+	+++
	D	+	++	++	+
	E	+++	+	+	+++


**Table 3:** Gene expression analysis showing the expression of α_5_, β_1_, β_3_, and β_5_ integrin subunits for 2D BMfMSCs as single cells in 2D (i); for BMfMSC-spheroids in 2D (ii); for 3D BMfMSCs as single cell in 3D (iii); and for BMfMSC-spheroids in 3D (iv).

(i) 2D BMfMSC single cell gene expression (*n* ≥ 6).				

Time point	Composition	Fold change relative to composition A		
		
		β_1_	β5	α_5_
Day 2	A	1	1	1
	B	0.62	0.68	1.30
	C	0.59	1.86	2.52
	D	16.46	3.87	13.5
	E	3.22	11.9	-
Day 4	A	1	1	1
	B	0.18	0.16	-
	C	0.20	0.15	0.50
	D	0.47	0.37	0.42
	E	-	4.64	-
Day 6	A	-	1	-
	B	-	0.27	-
	C	-	0.44	-
	D	-	1.50	-
	E	-	2.61	-

**(ii) 2D BMfMSC-Spheroid Gene Expression (*n* ≥ 6).**				

**Time point**	**Composition**	**Fold change relative to composition A**		
		
		**β_1_**	**β5**	**α_5_**

Day 2	A	1	1	1
	B	3.31	2.3	-
	C	6.29	7	-
	D	-	33.6	-
	E	3.22	4.8	1.33
Day 4	A	1	1	-
	B	1.33	1.26	-
	C	-	4.93	-
	D	3.48	5.22	-
	E	5.64	6.68	-
Day 6	A	1	1	1
	B	1.198	1	12.75
	C	1.1	0.72	-
	D	-	4.58	2.65
	E	-	4.88	-

**(iii) 3D BMfMSC-single cell gene expression (*n* ≥ 6).**				

**Time point**	**Composition**	**Fold change relative to composition A**		
		
		**β_1_**	**β_3_**	**β_5_**

Day 3	A	1	1	1
	B	14.4	-	29.06
	C	0.76	0.71	-
	D	-	-	24.11
	E	-	-	17
Day 6	A	1	-	1
	B	0.13	-	2.15
	C	-	-	0.06
	D	-	-	3.24
	E	-	-	-
Day 9	A	1	1	1
	B	1.15	1.59	2.12
	C	2.77	0.79	1.4
	D	11.98	-	12.51
	E	-	-	-
Day 12	A	1	1	1
	B	0.85	-	3.19
	C	1.37	-	1.55
	D	1.44	-	-
	E	-	-	-
Day 14	A	1	1	1
	B	1.3	0.24	0.64
	C	1.07	0.28	0.79
	D	2.55	2.46	2.36
	E	20.25	3.8	10.87

**(iv) 3D BMfMSC-spheroid gene expression (*n* ≥ 6).**					

**Time point**	**Composition**	**Fold change relative to composition A**			
		
		**β_1_**	**β_3_**	**β_5_**	**α_5_**

Day 3	A	1	1	1	1
	B	1.15	0.97	3.48	2.75
	C	0.65	0.76	1.05	1.15
	D	0.62	1.13	0.14	0.66
	E	8.08	12.61	20.651	14.97
Day 6	A	1	1	1	1
	B	1.05	0.79	3.14	3.2
	C	0.46	0.69	1.13	1.28
	D	0.48	1.01	0.34	0.89
	E	4.04	10.98	19.30	16.25
Day 9	A	-	-	-	1
	B	-	-	-	-
	C	-	-	-	0.99
	D	-	-	-	1.34
	E	-	-	-	0.52
Day 12	A	-	-	-	-
	B	-	-	-	-
	C	-	-	-	-
	D	-	-	-	-
	E	-	-	-	-
Day 14	A	-	-	-	1
	B	-	-	-	0.28
	C	-	-	-	0.17
	D	-	-	-	1.75
	E	-	-	-	5.06


## Discussion

Mesenchymal stem cells have been clinically tested for treating several disorders, including bone ailments, cardiovascular diseases, nervous system disorders, and autoimmune diseases (Parekkadan and Milwid, 2010; Hollweck et al., 2012). Although the reports from these clinical studies have had mixed results, there are some indications where MSCs have had a beneficial impact. For example, human patients receiving MSC transplants following MI have shown significant improvements in cardiac output parameters at early time-points (Chen et al., 2004). Ischemic cardiomyopathy patients receiving MSC grafts indicated increased functional capacity, better quality-of-life and improved ventricular remodeling (Hare et al., 2012). Two MSC-treated osteoarthritis patients demonstrated significant increase in total reduction in their visual analog scale (VAS) scores at 12 weeks post-treatment (Pak, 2011). Four early-stage systemic lupus erythematosus patients treated with allogeneic MSCs showed stable disease remission for 12–18 months (Sun et al., 2009). Six out of 10 spinal cord injured patients treated with MSCs measured improvement in motor function scores of the upper extremities and three of them reported improvement in their daily living activities (Park et al., 2012). MRI data for these patients also showed beneficial electrophysiological changes at 30 months follow-up post-intervention.

Human fMSCs may be a good source of stem cells for tissue repair in certain indications (O’Donoghue and Chan, 2006). In bone tissue engineering applications, fMSCs exhibited greater proliferation capacity, robust osteogenic potential and lower immunogenicity, as compared to adult MSCs (Zhang et al., 2010, 2012). Clinical experience using allogenic HLA-mismatched fMSC transplantation resulted in engraftment and differentiation into bone in a human female fetus with multiple intrauterine fractures, even though the recipient was immunocompetent (Le Blanc et al., 2005). After 10 months of transplantation, 7.4% of Y positive cells in the whole genome and 0.3% of XY positive cells in slides were identified in bone biopsy specimens and the bone histology revealed normal bone trabeculae. After 2 years, the child’s psychomotor development and growth were normal.

Pre-clinical experience with fMSCs in other indications have also demonstrated efficacy in treating tissue disorders. Airey et al. (2004) injected human fMSCs intraperitoneally into sheep fetuses and demonstrated successful engraftment of these cells into the hearts of fetal lambs *in utero*. They reported no differences in engraftment of hMSCs from adult bone marrow, fetal brain, and fetal liver. Chan et al. (2007) transplanted fMSCs into dystrophic mice at E14-E16 gestation and observed widespread, long-term engraftment with indication of myogenic differentiation of MSCs into skeletal and myocardial muscle as well as other cell types, including hepatocytes. The same group used fMSCs to demonstrate enhancement of phenotype in a mouse model of Type III Osteogenesis Imperfecta (OI) and found that donor cells were localized to bone and expressed osteoblast lineage genes and produced the extracellular bone structural protein osteopontin (Guillot et al., 2008).

The benefits of fMSC therapy notwithstanding, there are still several limitations that hinder efficacy, most notably cell survival and localized integration of the cell graft in the harsh environment of the diseased or injured tissue (Burdick et al., 2016). Hydrogels have been proposed as a carrier to deliver grafted cells while temporarily protecting them and localizing their therapeutic effects (Seliktar, 2012). However, the modulus of the hydrogels has to be carefully considered because it can affect stem cell renewal, structure, function and commitment to differentiation into target cells (Kerscher et al., 2016). Lv et al. (2015) elucidated the mechanism by which stem cells differentiate and grow in response to a substrate stiffness that mimics the physiological condition of the cells based on the observations that a hydrogel substrate with a Young’s modulus (E) that matches the stiffness of the native tissue ECM directs the stem cell differentiation toward that corresponding tissue lineage. Hydrogel substrates mimicking ECM properties of pancreas (*E* = 1.2 kPa), brain (*E* = 0.1 to 1 kPa), muscle (*E* = 8 to 17 kPa), bone tissue (*E* = 25 to 40 kPa), and cartilage (*E* = 3 kPa) direct MSCs to differentiate into beta cells, neurocytes, myoblasts, osteoblasts and chondrocytes, respectively. Several other studies reported similar findings when investigating how stem cell behavior corresponded to matrix stiffness, analyzing gene expression changes and intracellular signaling cascades emanating from mechanical cues from the culture substrate (Engler et al., 2006; Narayanan et al., 2014; Wang et al., 2014).

We studied the behavior of BMfMSCs (as single cells) or BMfMSC-spheroids under the influence of five different stiffness levels of PF hydrogels having storage shear modulus ranging from G’ = 120 Pa to 2,300 Pa. These values correspond to Young’s modulus values of *E* = 360 Pa to 6,900 Pa. Our results indicate that the modulus of the PF hydrogels, altered by varying the relative amount of PEG-DA crosslinker, affected gene expression. The response of the cells culminated as different cell morphologies and metabolic activities in both 2D and 3D environments. In a 2D environment, for example, there was a significant increase in metabolic activity of singe cell BMfMSCs in contrast to the BMfMSC-spheroids, where there was a significant decrease in the metabolic activity from day 2 to day 6. We also found that there was a minimum seeding density required for the BMfMSC single cell experiments to enable these outcomes (7,000 cells per well).

In the 3D environment, the results were more confounding. Single BMfMSCs encapsulated in PF hydrogels showed a significant decrease in metabolic activity from day 3 to day 9, yet BMfMSC-spheroids encapsulated in certain PF hydrogels behaved differently. For example, in composition A and composition C hydrogels, there was a significant decrease in metabolic activity from day 3 to day 9, whereas in composition D and composition E hydrogels, there was an increase in metabolic activity for the same time points. Interestingly, the composition B hydrogels provided a similar 3D culture platform for the growth of both single cells and spheroids; exhibiting the same metabolic activity pattern from day 3 to day 6. (i.e., the metabolic activity increased from day 3 to 6 and then showed a drastic decrease by day 9). Our results thus suggest that in addition to the modulus of the hydrogel and cell seeding density, the culture condition of single cells versus spheroids also influences the metabolism of the cells.

The different modulus of the PF hydrogels influenced morphological changes in the BMfMSCs, reflecting the dependency of morphogenesis on matrix modulus. For example, in 2D experiments, as the stiffness of the hydrogel increased, more protrusions of actin rich structures at the cell surface (i.e., lamellipodia) were observed for single cell cultures whereas more filopodia were observed for BMfMSC-spheroids at later time points of the study. Filopodia are actin filament bundles that protrude from lamellipodia to form spikes which can sense the ECM and direct cell migration. They serve as a type of mechanical sensor for migrating cells to identify suitable adhesion sites on neighboring cells (Xue et al., 2010). These protrusions also contribute to other cellular processes including wound healing, chemotaxis, adhesion, and neuronal growth cone formation (Mattila and Lappalainen, 2008). Filopodia can turn into lamellipodia in order to coordinate cell motility in response to surface topography (Collart et al., 2014). In this 2D study, BMfMSC-spheroids on composition B and composition D hydrogels form better cellular interconnections by exhibiting more filopodia at day 6. In contrast, the single cell BMfMSC cultures showed more filopodia at composition A, composition B and composition C, suggesting strong cellular interconnections as early as day 4. Another interesting finding was that when cell density was increased to 12,000 BMfMSCs per well in the single cell cultures, more lamellipodia were observed at the earlier time points in lower stiffness PF hydrogels (data not shown).

In 3D experiments, single cell BMfMSC cultures showed unique structural changes and exhibited only lamellipodia within the lowest (composition A) and highest stiffness hydrogels (composition D and composition E), whereas BMfMSCs encapsulated in composition B and composition C hydrogels exhibited both lamellipodia and filopodia at day 6. The encapsulation of BMfMSCs in moderate stiffness hydrogels could most likely sense their neighboring cells rapidly and form strong cellular interconnections. The BMfMSC-spheroids encapsulated in the PF hydrogels exhibited extensive lamellipodia and filopodia by day 9 in culture. At day 12, more filopodia were observed with an increase in stiffness of the hydrogels. The different levels of filopodia exhibited by the cells was identified across different time points. However, we found that certain modulus values of the PF hydrogel support higher degree of filopodia formation. Previous studies have elucidated that filopodia in MSCs may be involved in wound healing and tissue regeneration (Ryu et al., 2013; Mobasseri et al., 2017). Thus, by altering the stiffness of the hydrogel, BMfMSCs could be tuned to express more filopodia, thereby making them more amenable for specific tissue repair applications. Previous studies have also suggested the importance of filopodia activity for other functions (da Costa et al., 2012; Eke et al., 2017), including for the commencement of MSC differentiation into an osteoblastic lineage (Cassidy et al., 2014).

Several studies suggest that ECM guides cell adhesion, migration and encourages differentiation by activating certain integrin subunits (Dalby et al., 2014; Kshitiz Afzal et al., 2015). Hamidouche et al. (2009) demonstrated that activation of α_5_-integrin expression could direct MSCs toward osteoblastic differentiation. Schwab et al. (2013) showed that increased expression of α_5_ and α_V_ integrin subunits have positive effects on osteoblastic differentiation of MSCs. They also showed that the α_5_ integrin subunit has a positive role in guiding MSCs toward osteoblastic differentiation while β_3_ integrin subunit suppresses such differentiation. In our 2D single cell experiments, composition C and composition D hydrogels showed greatest expression of α_5_ integrin subunit compared to the composition A at the initial time point. However, the expression level decreased drastically and diminished at the later time points. In 2D spheroid experiments, composition B hydrogels showed a 12.7-fold increase in α_5_ integrin subunit compared to the composition A hydrogels at the later time points of the study, although the expression level was initially low. Considering the data from Schwab et al. (2013) and our observations on α_5_-integrin expression, with the complete absence of β_3_ integrin subunit in the 2D experiments, we speculate that these PF hydrogel formulations together with BMfMSCs could potentially be used for osteoblastic differentiation.

In our 3D single cell experiments, composition D and composition E hydrogels showed more than two to three times the expression levels of β_3_ integrin subunit compared to the composition A hydrogel at day 14. The 3D spheroid experiments showed that the composition E hydrogels have higher expression levels of β_3_ integrin subunit compared to composition E hydrogels at day 3 and day 4. Previous research determined that a medium stiffness substrate that promotes β_3_ integrin subunit expression can also mediate MSCs toward a myogenic differentiation pathway (Yu et al., 2013). Du et al. (2011) suggested that a soft matrix enhances β_1_ integrin subunit internalization, which in turn promoted endocytosis factors and resulted in the expression of neuronal genes in stem cells. Previous studies have stated that reduced β_1_ integrin activation could lead to keratinocyte differentiation (Lv et al., 2015). We found increased expression of β_1_ integrin subunit when BMfMSCs were encapsulated as single cells in the higher stiffness composition E hydrogels, but observed the absence of β_1_ integrin subunit expression when BMfMSCs were seeded as single cells in composition E hydrogels. Taken together, we speculate that the right combination of BMfMSC culture conditions (i.e., single cells or spheroids) and optimal modulus of the PF hydrogel could be designed to promote upregulation or suppression of β_1_ integrin subunit expression for lineage-specific differentiation to muscle, nerve or other tissue types.

In previous experiments using pericyte-derived stem cells and muscle-derived satellite cells as single cell cultures within optimized PF hydrogels, we did maximized differentiation to muscle lineages by fine tuning of the hydrogel modulus to approximately G’ = 200 Pa (Fuoco et al., 2012, 2014, 2015; Costantini et al., 2017). We speculate that the mid-stiffness PF hydrogels together with single cell BMfMSCs in 3D culture may likewise have optimal properties to support myogenic differentiation and related applications, although further studies would be required to verify this hypothesis.

### Limitations

We analyzed the metabolism and morphogenesis of BMfMSCs in or on hydrogels of varying stiffness based on correlations found in previous studies and our gene expression analysis. However, we did not evaluate the specific lineage determination of the cells. Future experiments must be performed to identify specifically how the different stiffness environments affect the lineage of the BMfMSCs as well as their proliferation and self-renewal. Further studies are also needed to optimize specific differentiation environments and media compositions, and to study the functional assays of transplanted cells to determine the ideal hydrogel for each specific clinical application. For example, BMfMSCs in the appropriate stiffness hydrogel could be cultured in myogenic differentiation medium for a particular time frame and could be tested for myogenic gene expression, as part of a complete strategy to enhance tissue regeneration following muscle injury. Further studies, including transplantation of hydrogels with differentiated cells into a myocardial repair model to examine their role and efficacy in improving the diseased state are also required to substantiate our hypotheses.

## Conclusion

Based on the results, different degrees of PF hydrogel stiffness influenced the distribution of F-actin filaments, metabolic activity of BMfMSCs, tube length and also the expression levels of several integrin subunits. The stiffness of the PF hydrogel could be altered by varying amounts of PEG-DA crosslinker in the hydrogel composition. We also found that cell seeding density is an important factor which promotes sufficient cell-to-cell contact, and that such interactions must take place before remodeling could occur in or on the PF hydrogels. Our findings provide valuable information for scaffold design for a wide range of applications and also suggest methods with which to optimize cell culture in responsive hydrogel scaffolds.

## Author Contributions

AR and SC designed the experiments, conducted the research, analyzed the data, supervised the research, and prepared the manuscript. MM and KL designed and performed the experiments, analyzed the data, and prepared the manuscript. MC, CM, JC, TK, and DS designed the experiments, analyzed the data, supervised the research, and prepared the manuscript.

## Conflict of Interest Statement

The authors declare that the research was conducted in the absence of any commercial or financial relationships that could be construed as a potential conflict of interest. The handling Editor declared a past co-authorship with one of the authors DS.
